# Triage Temperature and Timeliness of Sepsis Interventions in a Pediatric Emergency Department

**DOI:** 10.5811/westjem.47379

**Published:** 2025-11-26

**Authors:** McKenna Straus, John M Morrison, Racha Khalaf, Jamie Fierstein, Alexandra Miller, Diana Young, Elliot Melendez

**Affiliations:** * Johns Hopkins All Children’s Hospital, Office of Medical Education, St. Petersburg, Florida; † Johns Hopkins All Children’s Hospital, Division of Hospital Medicine, St. Petersburg, Florida; ‡ University of South Florida Morsani College of Medicine, Department of Gastroenterology, Hepatology and Nutrition, Tampa, Florida; § Johns Hopkins All Children’s Hospital Institute for Clinical and Translational Research, St. Petersburg, Florida; || Connecticut Children’s Hospital, Department of Pediatric Critical Care, Hartford, Connecticut

## Abstract

**Introduction:**

Fever as an indicator of infection is frequently used as an aid in triggering concern for sepsis in the emergency department (ED). Adults with sepsis presenting to the ED with a normal temperature have been shown to have delays in treatment and greater mortality. The association between temperature and timeliness of sepsis-related care in the ED remains poorly characterized in children. Our objective in this study was to measure the association between body temperature at the physiologic onset of sepsis and the time to initiation of antibiotic treatment and fluid bolus among children with clinically defined sepsis.

**Methods:**

We conducted a retrospective, cohort study of pediatric patients with sepsis presenting to the ED. Data collected from an existing quality improvement database were supplemented via chart extraction. We assessed body temperature at physiologic onset of sepsis (PO-S), the date and time when a patient first met clinical criteria for sepsis as defined by Goldstein et al.^1^ Our primary outcomes were time from PO-S and administration of antibiotics and fluid bolus. Secondary outcomes included maximum vasoactive-inotropic scores, need for extracorporeal membrane oxygenation (ECMO) within 30 days of presentation, presence and type of organ dysfunction, 30-day hospital- and intensive care unit (ICU)-free days, and mortality. We summarized and compared data by temperature group. Multivariable quantile regression was used to evaluate adjusted associations between body temperature and time to initiation of antibiotic treatment and fluid bolus.

**Results:**

Of 928 patients screened, 385 (41%) met inclusion criteria. Median time to antibiotic treatment did not differ between temperature groups at PO-S—≤ 36.0 °C: median (IQR) 48.5, (41.3–104.8); 36.1–37.9 °C: median, 95.5, (41.3–104.8;), and ≥ 38.0 °C: median 84, 45–151; (*P* = .24). Median time to fluid bolus administration also did not differ between temperature groups at PO-S—≤ 36.0 °C: median 39, (20.8–65.8); 36.1–37.9 °C: median, 42.5 (21.3–86.3); and ≥ 38.0 °C: median, 54 (29–84); (*P* =.07). In addition, mortality differed by temperature at PO-S (≤ 36.0 °C: 1/22 (4.5%); 36.1–37.9 °C: 4/80 (5.0%); and ≥. 38.0 °C: 3/283 (1.1%), (*P* = .04); as did organ dysfunction at 72 hours: ≤. 36.0 °C: 15/22 (68.2%); 36.1–37.9 °C: 43/80 (53.8%), ≥ 38.0 °C: 74/283 (26.1%); (*P* < .001) and median (IQR) 30-day ICU- and hospital-free days—≤ 36.0 °C: median, 24, (20,−26.8); 36.1–37.9 °C: median, 28 (24.8–30), ≥ 38.0 °C: median, 30 (27–30), (*P* < .001); and at ≤. 36.0°C: median, 22, (17–25); 36.1–37.9 °C: median, 24 (17.8–27); ≥ 38.0 °C: median, 25 (20, 27), (*P* = .04), respectively. We did not observe an association between temperature and median time to antibiotic administration (β: 2.5, 95% CI, −4.2 to 9.1, *P* = .50) or first fluid bolus administration (β: 1.7, 95% CI, −1.4 to 4.8, *P* = .30).

**Conclusion:**

Time to fluid bolus administration and time to antibiotic administration did not differ statistically by temperature from physiological onset of sepsis. Children presenting with hypothermia (≤ 36.0 °C) had worse outcomes.

## INTRODUCTION

Sepsis, defined as a dysregulated host response to an infecting pathogen associated with life-threatening organ dysfunction, accounts for approximately 4,500 pediatric mortalities annually in the United States.^2^ Pediatric mortality rates attributable to sepsis range from 4–25%, with many of those deaths occurring within the initial 48–72 hours of presentation.^3^,^4^ The emergency department (ED) often serves as the first point of contact for septic children.^5^ Timely intervention with early fluid resuscitation and antibiotic administration in the ED improves sepsis-related morbidity and mortality by up to 50%.^6^ Previous studies have demonstrated that delayed therapy is associated with increased mortality and prolonged organ failure.^2^,^7^–^10^ Accordingly, early recognition of sepsis is essential to timely clinical intervention.

In the setting of systemic infection, a host immune-mediated response results in the rising of the body temperature set point via the hypothalamus.^11^ Hypothermia has also been shown to be associated with serious bacterial infections.^12^ Sepsis screening tools use clinical data, including body temperature, to recognize patients at risk for sepsis and can improve the timeliness of subsequent clinical intervention.^13^ Although no universally accepted sepsis-screening tool exists, an important data element common to pediatric recognition tools is the history of an abnormal temperature.^13^ Research in adults with severe sepsis and septic shock has shown that elevated body temperature greater than 37 °C in the ED is associated with reduced time to antibiotic, but not fluid bolus administration, likely due to a heightened awareness of potential infection.^14^

The impact of normal or low body temperatures on the timeliness of sepsis care in the ED is less understood. Park et al showed that in comparison to adult septic patients presenting with hyperthermia, patients presenting with normothermia (36–38 °C) had more than twice the risk of in-hospital mortality and lower compliance with sepsis bundles.^15^ The association between temperature at the presentation of illness and the administration of timely antibiotics and fluid boluses within recommended time frames remains poorly characterized. Accordingly, we aimed to investigate the association between body temperature at the physiologic onset of sepsis (PO-S) and timeliness to initiation of antibiotics and fluid bolus in children presenting to the ED with clinically suspected sepsis. We hypothesized that body temperature is associated with both the time to initiation of antibiotics and administration of first fluid bolus.

## METHODS

### Study Population and Design

We conducted a single-center, retrospective cohort study of pediatric patients 0–21 years of age with clinically defined sepsis who presented to the ED from January 1, 2017–February 28, 2021. Our ED is located within a 259-bed quaternary-care, freestanding children’s hospital with an annual volume of approximately 30,000 visits per year. We extracted data from an existing institutional quality improvement database of patients with sepsis who contributed data to the Children’s Hospital Association Improving Pediatric Sepsis Outcomes (IPSO) Collaborative.^16^ The IPSO Collaborative is a multi-institutional quality improvement initiative with the intent to reduce sepsis-related mortality and improve patient outcomes through early identification and intervention. Additional supplemental data specific to our study were extracted from our local electronic health record. We included patients meeting at least one operational definition of sepsis using the IPSO intention-to-treat algorithm in the institutional database.^17^

**Population Health Research Capsule**

What do we already know about this issue?

*Fever triggers concern for sepsis. Adults with sepsis presenting with normothermia have been shown to have delays in treatment and greater mortality.*

What was the research question?

*Is there an association between body temperature and time to sepsis treatment for children presenting to the emergency department?*

What was the major finding of the study? *There was no association between temperature and time to antibiotic (CI, −4.1 to 9.1, P =.50) or fluid bolus (CI, −1.4 to 4.8, P =.30).*

How does this improve population health?

*Body temperature should be used in conjunction with other clinical signs of sepsis to promptly treat children in the ED.*

Patients were considered to have sepsis if the patient received an intravenous antibiotic, ≥ 2 fluid boluses (or one fluid bolus plus initiation of a pressor) within six hours of presentation and a blood culture was obtained within 72 hours of presentation, plus at least one of the following: documentation of *International Classification of Diseases, 10th Rev*, codes (ICD-10) for sepsis (R65.20/R65.21); positive institutional sepsis screen; use of institutional septic shock order set; intensive care unit (ICU) admission; or documentation of sepsis-related ICD-10 codes (A02.1, A20.7, A21.7, A22.7, A24.1, A26.7, A32.7, A39.2–4, A40.0–3, A40.8–9, A41.01–2, A41.1–4, A41.50–3, A41.59, A41.81, A41.89, A41.9, A42.7, A54.86, B00.7, B37.7). We excluded patients who were originally treated at an outside facility or were not treated within the ED. Additional patients were excluded if they had missing data on body temperature at triage or timing of medical intervention (fluid bolus and/or antibiotic administration). This study was approved by our Institutional Review Board (IRB 00287405). We followed criteria 1–5, 9–12 from Methods of Medical Record Review Studies in Emergency Medicine Research. The abstractor was not blinded to the hypothesis but was blinded to the outcome.^18^

### Exposure Measurements

The primary study exposure was body temperature in degrees Celsius at time of patient’s PO-S. During this study, our organization was involved in the IPSO C-ollaborative where an alert occurred when a child met criteria for concern for sepsis if ≥ 3 physiologic criteria associated with sepsis occurred. The time the concern for sepsis occurred was consider the physiological onset of sepsis if the alert led to obtaining a blood culture and initiating intravenous antibiotics, and if at least two fluid boluses (or fluid bolus with initiation of vasopressor) were given.^1^,^16^

### Outcome Ascertainment

The first primary outcome was the difference in minutes between PO-S and administration of antibiotics. The second primary outcome was the time to administration of first fluid bolus. Secondary outcomes included maximum vasoactive-inotropic scores,^19^ need for extracorporeal membrane oxygenation (ECMO) within 30 days of presentation, presence and type of organ dysfunction, 30-day hospital and intensive care unit (ICU)-free days, and mortality. We defined 30-day ICU- and hospital-free days as the number of days within the first 30 days after admission that a patient was alive and not admitted to either the hospital or ICU. We calculated this composite measure of both mortality and duration of hospitalization, as opposed to length of stay, to eliminate bias of early mortality.

### Covariate Measurements

Data on patient clinical characteristics included source of infection (as determined by positive culture or as described in the discharge summary), comorbidities that may place a child at a higher risk of infection (malignancy, asplenia, bone marrow or solid organ transplant, immunocompromise, technology dependent), presence of a central venous catheter, vasoactive agent initiation (where applicable), duration of hospitalization, and disposition. We calculated vasoactive-inotropic scores based on the medical administration record in the EHR. Additional data manually extracted from the EHR included patient demographics, medical comorbidities, and laboratory values (white blood cell count, C-reactive protein, erythrocyte sedimentation rate, and lactate).

### Statistical Analyses

For descriptive purposes, we categorized temperature as ≤ 36.0 °C, 36.1–37.9 °C, and ≥ 38.0°C. Patient demographic and clinical characteristics were summarized across body temperature groups at PO-S with medians and interquartile ranges, and frequencies and percentages for quantitative and categorical variables, respectively. We compared data across temperature groups using Kruskal-Wallis tests for quantitative variables and chi-square or Fisher exact test for categorical variables, as appropriate. When significant differences (< .05) across groups were observed with the omnibus test, we performed post-hoc pairwise analyses with a Bonferroni correction. Density and Q-Q plots suggested skewedness and non-constant variance in the outcomes across body temperature. Given these considerations and the a priori hypothesis asserting an inverted “U”-shaped association between the primary exposure and outcomes, multivariable quantile linear regression models with robust standard errors were fitted to evaluate the adjusted relationship between body temperature at PO-S and time to treatment with either antibiotic and/or fluid bolus at the 10th, 25th, 50th, 75th and 90th percentiles. For multivariable modeling, we analyzed temperature as a continuous variable. Beta coefficients were calculated and reported along with corresponding robust standard errors.

We adjusted models for sex at birth, age, race and ethnicity, number of medical comorbidities (0, 1, ≥. 2), and sepsis screening score (< 3, ≥ 3). Sepsis scores ≥ 3 indicate the patient may be at risk for sepsis with triggering of sepsis-specific bundle care. Sepsis scores, although containing body temperature, were controlled for because clinician intervention may have been secondary to elevated scores even in the absence of fever. We considered two-sided *P*-values < .05 statistically significant. With a given sample size of 385 patients and an α level of .05, the study had 90% power to detect a medium effect size using multivariable quantile regression modeling the association between temperature and primary outcomes with adjustment for seven additional covariates. Analyses were performed with Stata/SE v17.1 (StataCorp, LLC, College Station, TX), and we calculated power estimates with PASS Power Analysis and Sample Size software v16 2018 (NCSS, LLC, Kaysville, UT).

## RESULTS

### Patient Population

**Figure 1:**
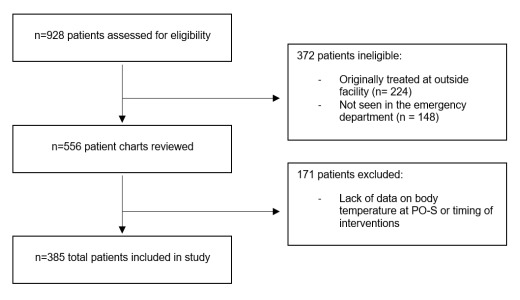
CONSORT diagram of patient inclusion in a study of the association of body temperature at physiologic onset of sepsis and timeliness of sepsis-related interventions. *PO-S*, physiologic onset of sepsis.

Of 928 patients we assessed for eligibility within the quality improvement database, 543 patients were ineligible or excluded. Overall, 385 patients were included in the final analysis (Figure 1). Demographic and clinical characteristics according to patient body temperature at PO-S are summarized in Table 1. Twenty-two patients (5.7%) had body temperatures ≤ 36.0 °C, while eight (20.8%) had temperatures 36.1–37.9 °C, and 283 (73.5%) had temperatures ≥ 38.0 °C. The distribution of oncologic comorbidities differed statistically by temperature group (*P* = .03). Patients with body temperature of 36.1–37.9 °C had the highest proportion of oncologic comorbidities, whereas zero patients with lower body temperatures of ≤ 36.0 °C had an oncologic comorbidity. No statistical differences were observed in the distribution of sex, race, ethnicity, or high-risk conditions.

### Sepsis Treatment-related Outcomes

**Figure 2:**
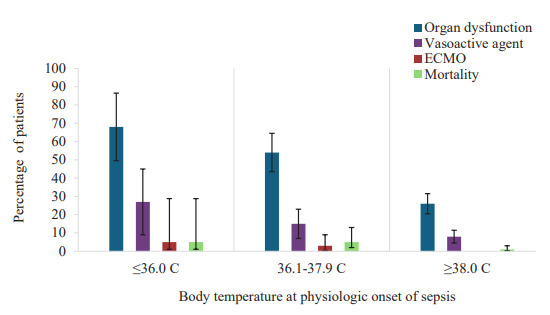
Sepsis-related outcomes stratified by body temperature categories of patients presenting to a pediatric emergency department in a study of the association of temperature and timeliness of sepsis-related interventions. The frequency of each outcome differed between temperature groups (*P* < .05 for all). *ECMO*, extracorporeal membrane oxygenation.

There were no statistical differences in time to antibiotic treatment or time to fluid bolus administration by temperature group as seen in Table 2. In comparison to patients with higher body temperatures, patients with temperature ≤ 36.0 °C had higher mortality rates (*P* = .04), fewer 30-day ICU-free days (*P* <.001), fewer 30-day hospital-free days (*P* = .04), higher proportions of organ dysfunction at 72 hours (*P* < .001), respiratory dysfunction (*P* = < .01), higher proportions of vasoactive agent use within 24 hours (*P* = <.01), and ECMO (*P* = .02) within 30 days of PO-S (Figure 2).

### Multivariable Analyses

In multivariable models measuring the association between body temperature at PO-S and time to antibiotic and time to fluid bolus, there was no detectable association between the temperature group and either outcome (Tables 3, 4). However, faster administration of both antibiotic and fluid bolus was seen with patients who had medical comorbidities and elevated sepsis scores. No other significant associations were observed for either primary outcome.

## DISCUSSION

In our study of children presenting to a pediatric ED receiving sepsis-specific care, body temperature at PO-S was not associated with time to initiation of antibiotic treatment or time to initiation of fluid resuscitation. Additionally, patients with triage temperature ≤ 36.0 °C more often received vasoactive agents or ECMO and experienced more frequent end-organ dysfunction and mortality. The influence of temperature at PO-S and timeliness to intervention remains unclear.

Providing timely, bundled sepsis care inclusive of prompt fluid resuscitation, blood culture acquisition, and administration of antibiotics in the ED decreases sepsis-related morbidity and mortality.^20^–^22^ Because provision of the entire bundle appears necessary for improvement in outcomes, there is a call for quality-based efforts that use targeted interventions focused on standardized recognition algorithms and team-based strategies to deliver life-saving care.^7^,^23^ Many sepsis recognition algorithms focus on the presence of an abnormal temperature as a non-specific indicator for potential sepsis. Fever is a common presenting symptom for children in the ED (the vast majority of whom do not have clinical sepsis), and the absence of an abnormal temperature may influence the timeliness of delivery of bundled sepsis care.

We detected no clinically relevant differences between body temperature at PO-S and time to initiation of antibiotics or fluid bolus. This contrasts with previous studies that showed normothermia in the setting of sepsis within the adult population was associated with lower compliance with timely sepsis interventions and higher rates of mortality.^14^,^15^ While fever is a common presenting symptom in EDs for pediatrics, it is far less common among adults.^24^ This difference in populations, may, in part, contribute to the timeliness of interventions, as the clinical suspicion for serious infection may be greater among adults presenting with fever.

As opposed to temperature at PO-S, we identified several other factors associated with timeliness of sepsis care. An elevated institutional sepsis screening score or having medical comorbidities was associated with more timely administration for both antibiotics and fluid bolus. At our institution, a patient with a sepsis score ≥ 3 triggers a multidisciplinary, sepsis-specific response including physician notification through the electronic health record. This is intended to prompt timely management of sepsis using care bundles. Children with ≥ 1 comorbidity are at higher risk for mortality secondary to sepsis and may prompt physicians to intervene sooner.^25^ In addition, our institution has a clinical practice guideline guiding antibiotic administration and acquisition of a blood culture within one hour for children presenting with fever and who have a history of bone marrow transplant without full engraftment and recovery or ongoing oncologic treatment. In these scenarios, it is likely that clinical context and institutional care pathways have a greater impact on timeliness of sepsis interventions than temperature alone.

In our study, we intentionally evaluated for disparities in timeliness of sepsis care between racial and ethnic groups. In other settings and conditions, Black patients are at higher risk of receiving a less urgent triage score, Black and Hispanic children are less likely to receive diagnostic imaging in the ED, and Black children are less likely to receive analgesia in the setting of appendicitis.^26^–^28^ Prior studies have suggested that implementation of electronic alerts and clinical pathways may reduce racial differences.^29^,^30^ Although not a primary explanatory variable, we did not detect an association between race or ethnicity and timeliness to sepsis interventions. Nonetheless, future studies involving the careful and deliberate evaluation of the impact of systemic and implicit biases on the timeliness of sepsis-related care for under-represented and marginalized groups are essential.

We also investigated whether clinically relevant outcomes monitored by the IPSO Collaborative differed between temperature groups. The prevalence of patients requiring vasoactive agents within 24 hours, use of ECMO within 30 days, and development of end-organ dysfunction within 72 hours differed between temperature groups and were more common in those presenting with temperatures ≤ 36.0 °C. Similarly, 30-day ICU-free days and mortality differed between groups, with hypothermic patients having fewer ICU-free days and higher mortality compared to those with normal or elevated temperatures. Our results are consistent with other studies suggesting association between hypothermia and worse outcomes in the setting of critical illness. Previously, hypothermia has been associated with increased mortality in both children and adults with sepsis.^31^–^35^ In other studies, hypothermia within 24 hours of sepsis diagnosis was associated with immune dysfunction as well as increased incidence of organ failure and disseminated intravascular coagulation.^36^–^37^

## LIMITATIONS

This study had several limitations. Due to its retrospective nature, we were unable to establish a causal association between temperature at PO-S and sepsis intervention and clinical outcomes. During the time in which our data was collected, our institution was involved in QI initiatives to improve sepsis care and, therefore, results may be biased and not reflective of all centers caring for children presenting with possible sepsis. It is possible sepsis was more quickly recognized and with prompter initiation of treatment secondary to the initiative. Additionally, with the primary predictor as temperature category and with such a small number of hypothermic patients, the study is underpowered to attempt to understand why patients with hypothermia did not receive more timely care. One theory is that with aggressive sepsis management processes linked to fever, in the absence of fever, clinicians may be focused on managing hypothermia and less on achieving timely intervention with the concern for sepsis. In addition, we conducted a secondary analysis of an existing database and, therefore, we were limited to the definitions, including sepsis and PO-S, defined previously by its creators.

We were also limited to the information in the EHR for identification of PO-S, which may have resulted in misclassification of cases with sepsis or exclusion of patients that should have ultimately been included. All patients in this study were included based on an intention to treat sepsis; however, not all patients were ultimately found to have an identifiable bacterial source. In our population, 14% of patients had a positive blood culture, 10% had a positive urine culture, and 2% had a positive cerebrospinal fluid culture. Because the data were collected from a single center with local institutional practices regarding bundled sepsis care, including the use of sepsis scoring triggering prompt action, the generalizability of our findings may be limited.

Furthermore, there may be additional confounders that would influence the timeliness of sepsis care such as ED patient volumes as well as individual clinician characteristics such as years of experience and tendencies to recognize and treat patients with suspected sepsis. As there is no validated severity of illness score for children in the ED, we could not reliably control for severity of illness that may have influenced both timeliness of care or secondary outcomes. However, we did include markers of severity such as hypotension on arrival that are a surrogate for shock and risk for greater severity of illness. Finally, body temperature was likely obtained via a variety of methods, and this was not standardized or recorded consistently, which may result in misclassification of patients regarding temperature at PO-S.

## CONCLUSION

No significant association between body temperature at PO-S and time to initiation of antibiotic treatment or fluid bolus was detected. Worse outcomes were observed among pediatric patients presenting with hypothermia (≤ 36.0 °C), but faster intervention among this group was not seen, highlighting a need for an improved clinical approach. Abnormal body temperature alone was not associated with faster intervention. However, patients with elevated sepsis scores, which include body temperature, received faster administration of both fluid bolus and antibiotics, suggesting that temperature is an important clinical factor that should be used in conjunction with other signs of sepsis. Further studies are needed to understand the influence of body temperature at time of triage on timeliness of sepsis related interventions.

## Figures and Tables

**Table 1:** Demographic and clinical characteristics of patients by body temperature at physiologic onset of sepsis presenting to a pediatric emergency department in a study of the association of temperature and timeliness of sepsis-related interventions.

	≤ 36.0 °C (n=22)	36.1–37.9 °C (n=80)	≥ 38.0 °C (n=283)	P-value
Sex at Birth, n (%)				
Female	9 (40.9)	40 (50.0)	130 (45.9)	.70
Race, n (%)				
White	15 (68.2)	49 (61.3)	168 (59.4)	.97
Black	5 (22.7)	16 (20.0)	65 (23.0)	
Asian-American	0 (0.0)	1 (1.3)	5 (1.8)	
American Indian/Alaska Native	0 (0.0)	0 (0.0)	2 (0.7)	
Multiracial	1 (4.5)	1 (1.3)	7 (2.5)	
Unknown	1 (4.5)	13 (16.3)	36 (12.7)	
Ethnicity, n(%)				
Hispanic/Latino	19 (86.4)	63 (78.8)	222 (78.4)	.87
Non-Hispanic/Latino	3 (13.6)	14 (17.5)	54 (19.1)	
Unknown	0 (0.0)	3 (3.8)	7 (2.5)	
High-risk Conditions Present, n (%)				
Malignancy	0 (0.0)	14 (17.5)	28 (9.9)	.03
Asplenia	0 (0.0)	5 (6.3)	14 (4.9)	.63
BMT	0 (0.0)	4 (5.0)	7 (2.5)	.35
Indwelling line/catheter	2 (9.1)	15 (18.8)	50 (17.7)	.62
Solid organ transplant	1 (4.5)	7 (8.8)	17 (6.0)	.70
Severe intellectual disability	7 (31.8)	20 (25.0)	77 (27.2)	.81
Immunocompromised	15 (68.2)	54 (67.5)	188 (66.4)	.97
Technology dependent	7 (31.8)	26 (32.5)	92 (32.5)	>.99
≥ 2 High-risk Conditions	7 (31.8)	39 (48.8)	127 (44.9)	.37
Severe Sepsis Selection Criteria, n (%)				
Positive sepsis screen (with treatment)	12 (54.5)	19 (23.8)	160 (56.5)	<.001
Positive huddle for severe sepsis	0 (0.0)	0 (0.0)	1 (0.4)	
Severe sepsis order set used (with treatment)	3 (13.6)	5 (6.3)	7 (2.5)	
ICU admission (with treatment	6 (27.3)	20 (25.0)	33 (11.7)	
Lactate (with treatment)	0 (0.0)	3 (3.8)	15 (5.3)	
Standalone ICD codes	1 (4.5)	3 (3.8)	5 (1.8)	
Other sepsis ICD-10 (with treatment)	0 (0.0)	30 (37.5)	62 (21.9)	
Organ System with Known Comorbidity, n(%)				
No comorbidities	11 (50.0)	24 (30.0)	102 (36.0)	.21
Neurologic	9 (40.9)	20 (25.0)	77 (27.2)	.32
Cardiovascular	1 (4.5)	7 (8.8)	17 (6.0)	.70
Pulmonary	7 (31.8)	17 (21.3)	57 (20.1)	.44
Gastrointestinal	9 (40.9)	25 (31.3)	93 (32.9)	.74
Nephrological	2 (9.1)	5 (6.3)	27 (9.5)	.72
Rheumatologic	0 (0.0)	1 (1.3)	5 (1.8)	> .99
Endocrinologic	0 (0.0)	4 (5.0)	23 (8.1)	.36
Other	0 (0.0)	7 (8.8)	15 (5.3)	.30
Multiple Comorbidities	9 (40.9)	34 (42.5)	105 (37.1)	.83
Source of Infection, n (%)				
No infection source identified	19 (86.4)	61 (76.3)	224 (79.2)	.74
Pulmonary	13 (59.1)	29 (36.3)	120 (42.4)	.19
Urinary	2 (9.1)	7 (8.8)	38 (13.4)	.50
Skin and soft tissue	0 (0.0)	6 (7.5)	18 (6.4)	.52
Catheter-associated	1 (4.5)	4 (5.0)	20 (7.1)	.87
Bacteremia without specific site	1 (4.5)	3 (3.8)	3 (1.1)	.12
Meningitis	0 (0.0)	1 (1.3)	14 (4.9)	.25
Other	0 (0.0)	3 (3.8)	11 (3.9)	>.99

Percentages may not add to exactly 100% due to rounding.

*BMT, bone marrow transplant; ICD-10*, International Classification of Diseases, 10^th^ Rev; *ICU*, intensive care unit.

**Table 2:** Sepsis treatment-related outcomes by body temperature at physiologic onset of sepsis of patients presenting to a pediatric emergency department in a study of the association of temperature and timeliness of sepsis-related interventions.

	≤36.0 °C n=22	36.1–37.9 °C n=80	≥ 38.0 °C n=283	*P*-value	≤ 36.0 °C vs 36.1–37.9 °C Adjusted *P*-value^2^	≤36.0 °C vs ≥ 38.0 °C Adjusted *P-*value^2^	≥ 38.0 °C vs 36.1–37.9 °C Adjusted *P-*value^2^
Time to antibiotic treatment (minutes), median [IQR]	48.5 [41.3, 104.8]	95.5 [41.3, 104.8]	84 [46, 151]	.24	.39	.39	>.99
Time to first fluid bolus (minutes), median [IQR]^1^	39.0 [20.8, 65.8]	42.5 [21.3, 86.3]	54.0 [29, 84]	.07	>.99	.24	.27
Vasoactive agent within 24 hours, n (%)	6 (27.3)	12 (15.0)	23 (8.1)	<.01	.54	.01	.20
ECMO within 30 days, n (%)	1 (4.5)	2 (2.5)	0 (0)	.02	>.99	<.001	.02
Blood culture positive, n (%)	4 (18.2)	9 (11.3)	41 (14.5)	.62	>.99	>.99	>.99
Urine culture positive, n (%)	2 (9.1)	8 (10.0)	28 (9.9)	.95	>.99	>.99	>.99
CSF culture positive, n (%)	0 (0)	1 (1.3)	8 (2.8)	>.99	-	-	>.99
Organ dysfunction within 72 hours, n (%)	15 (68.2)	43 (53.8)	74 (26.1)	<.001	.68	<.001	<.001
Cardiovascular dysfunction	5 (22.7)	15 (18.8)	31 (11.0)	.68	>.99	>.99	>.99
Respiratory dysfunction	12 (54.5)	19 (23.8)	23 (8.1)	<.01	.25	<.01	.48
Neurological dysfunction	0 (0)	1 (1.3)	4 (1.4)	.81	-	-	>.99
Hematologic dysfunction	4 (18.2)	10 (12.5)	13 (4.6)	.57	>.99	>.99	>.99
Renal dysfunction	5 (22.7)	3 (3.8)	12 (4.2)	.05	>.99	.72	>.99
Hepatic dysfunction	8 (36.4)	24 (30.0)	36 (12.7)	.75	>.99	.32	.29
Multiple organ dysfunction	9 (40.9)	18 (22.5)	24 (8.5)	.12	.96	.09	.60
Mortality, n (%)	1 (4.5)	4 (5.0)	3 (1.1)	.04	>.99	.50	.07
ICU-free days, median [IQR]^1^	24.0 [20, 26.8]	28.0 [24.8, 30]	30.0 [27, 30.0]	<.001	<.01	<.001	.02
Hospital-free days, median [IQR] 1	22.0 [17, 25]	24.0 [17.8, 27]	25.0 [20, 27]	.04	>.99	.12	.27

1 IQR is reported as [25^th^ percentile, 75^th^ percentile]

2 A Bonferroni correction was applied to account for multiple comparisons between groups.

*CSF*, cerebrospinal fluid; *ECMO*, extracorporeal membrane oxygenation; *ICU*, intensive care unit.

**Table 3:** Adjusted association between temperature at physiologic onset of sepsis and time to antibiotic administration of patients presenting to a pediatric emergency department in a study of the association of temperature and timeliness of sepsis-related interventions.

N = 385	β Coefficient	95% CI	*P*-value
Temperature at PO-S (°C)	2.5	−4.2 to 9.1	.50
Sex at Birth			
Female	Ref.		
Male	−15.3	−34.6 to 3.9	.10
Age (years)	0.1	−1.4 to 1.7	.90
Ethnicity			
Hispanic/Latino	−17.1	−46.3 to 12.1	.30
Non-Hispanic/Latino	Ref.		
Unknown	−10.7	−73.1 to 51.7	.70
Race			
AIAN/Asian/Native Hawaiian/Pacific Islander/Multiracial	−17.6	−65.2 to 30.0	.50
Black	−12.0	−36.5 to 12.6	.30
Unknown	−5.1	−38.2 to 28.0	.80
White	Ref.		
Sepsis Score			
< 3	Ref.		
≥ 3	−57.3	−82.0 to −32.6	<.001
Not Reported	−28.8	−56.4 to −1.3	.04
Hypotension			
Hypotension within 15 minutes of triage	−2.8	−35.8 to 30.2	.90
Medical Comorbidities			
None	Ref.		
1	−53.0	−78.1 to −27.9	<.001
≥ 2	−26.7	−51.5 to −1.9	.04

*AIAN*, American Indian and Alaska Native*; PO-S*, physiologic onset of sepsis*; Ref*, reference variable

**Table 4:** Adjusted association between temperature at physiologic onset of sepsis and time to first fluid bolus of patients presenting to a pediatric emergency department in a study of the association of temperature and timeliness of sepsis-related interventions.

N = 385	β Coefficient	95% CI	P-value
Temperature at PO-S (°C)	1.7	−1.4 to 4.8	.30
Sex at Birth			
Female	Ref.		
Male	−9.3	−18.2 to −0.3	.04
Age (years)	−0.2	−0.9 to 0.5	.60
Ethnicity			
Hispanic/Latino	−4.3	−17.9 to 9.2	.50
Non-Hispanic/Latino	Ref.		
Unknown	4.6	−24.4 to 33.6	.80
Race			
AIAN/Asian/Native Hawaiian/Pacific Islander/Multiracial	3.9	−18.2 to 26.0	.70
Black	1.0	−10.4 to 12.4	.90
Unknown	2.5	−12.9 to 18.0	.70
White	Ref.		
Sepsis Score			
< 3	Ref.		
≥ 3	−13.5	−25.0 to −2.0	.02
Not Reported	−18.9	−31.7 to −6.1	<.01
Hypotension			
Hypotension within 15 minutes of triage	−15.3	−30.7 to 0.03	.05
Medical Comorbidities			
None	Ref.		
1	−22.9	−34.6 to −11.3	<.001
≥ 2	−11.1	−22.6 to 0.5	.06

*AIAN*, American Indian and Alaska Native; *PO-S*, physiologic onset of sepsis; *Ref*, reference variable
